# A Case of Renal Tumor

**Published:** 1873-09

**Authors:** 

**Affiliations:** Monroe County N. Y.


ART IV.—*A Case of Renal Tumor.* By DR. DENMAN, Monroe County N. Y.

F. C., the subject of this paper, was the child of healthy Irish parents, and was considered a healthy child until six months old, when his mother discovered a "lump" in his right side, below the short ribs.

She describes it as being about 2½ or 3 inches in diameter and nearly round at that time, and quite firm. A few weeks after this, and before the parents had consulted a physician, the child had an attack of hæmaturia, quite excessive at first, but subsiding in a couple of days. Occasionally after this he would pass clots of blood, but never had another attack of hæmaturia. He continued to grow finely until eight or nine months old, the tumor gradually increasing in size in the meantime. After this time, if he grew at all, it was more slowly; the limbs lost their plumpness, and the skin lost its ruddy, healthful appearance, and notwithstanding the administration of remedies prescribed by various practitioners, the tumor continued to increase in size.

The functions of the kidneys, as well as the other abdominal viscera, appeared to be properly performed. The urine, however, was highly colored, and gave forth a strong odor continually, and there was but a small quantity retained at a time by the bladder, much of the time during the day it was dribbling away as it was secreted.

I first saw the child when he was about twenty-two months old. His muscular system at this time was greatly emaciated, and he presented a general chlorotic appearance. At this time he had ceased to walk, and his feet and legs were infiltrated with serum. His appetite was very good, and he enjoyed sitting in his chair or wagon, or lying upon the floor playing with his toys.

His abdomen was very greatly distended; its walls were very tense and uniform in shape, the base of the thorax, however, being greatly distended, this giving the abdomen and thorax together a conical form.

There was general dullness upon percussion over the abdomen, but no fluctuation.

The surface was smooth, and the skin marked with a net-work of veins.

He lived four months after this, gradually becoming more emaciated and more and more helpless, owing to the increased size of the tumor, and probably, also, to a constant loss of strength. The last few days of his life he had severe paroxysms of coughing, his mother thinking he had the whooping cough. His appetite continued good to the last, and he died in a paroxysm of coughing while eating his breakfast, on the morning of the 5th of last September, aged two years and about two months.

Twenty-eight hours after death, assisted by Drs. Starkey, Behan and Gandy. General appearance of the cadaver much the same as that presented by the body four months before, with the exception of a greater emaciation of the muscles, and an increase in the size of the abdomen, the walls of which were very thin and tense. Upon exposing the tumor, it was found adherent to the peritoneum at a couple of points, and to the colon and small intestines quite generally.

It occupied the whole of the right side and front of the abdominal cavity, crowding the intestines to the left side of the spine. The bladder was not examined. The other abdominal viscera were only examined in situ, but presented a normal appearance. The right kidney was absent; the tumor weighed ten pounds; was cylindrical in form, about seven inches in diameter and eleven inches in length, rounded off at either end. Upon cutting into it the interior presented a cheesy appearance.

Having the tumor in the office of Prof. Moore, of this city, for examination, his son E. M. Moore, Jr., discovered an ureter on its surface, which upon being dissected up to its origin was found to be pervious and opening; a part into a pelvis which communicated with a portion of normal and apparently healthy kidney; and the remaining part into another pelvis, or what appeared like one, which communicated with the tumor by several appertures, which were extended into deep cells in the substance of the tumor.

The microscope showed the white cheese-like substance of the tumor to be cellular.

I have not been able to learn that there was any test made for albumen in this case. This is to regretted, as it might have been a guide had albumen been found, to the cause of the pathological change.

Rokitansky says:—We find fibrous masses of various extent and shape developed in the products left by inflammation and Bright's disease.

Dr. Bright, in his volume on abdominal tumors, gives twelve cases in which they were connected with the kidney, one of which was cystic; six were puriform collections, and the remaining five were malignant, all occurring in adults.

Dr. Wood speaks of one case of puriform collection weighing sixty-eight pounds.

Reports of cases of this kind, are, I think, quite rare.

